# Influence of benthic currents on cold-water coral habitats: a combined benthic monitoring and 3D photogrammetric investigation

**DOI:** 10.1038/s41598-020-76446-y

**Published:** 2020-11-10

**Authors:** Aaron Lim, Andrew J. Wheeler, David M. Price, Luke O’Reilly, Kimberley Harris, Luis Conti

**Affiliations:** 1 School of Biological, Earth and Environmental Science, Environmental Research Institute, University College Cork, Cork, Ireland; 2 Irish Centre for Research in Applied Geosciences, Marine and Renewable Energy Institute, University College Cork, Cork, Ireland; 3 Ocean and Earth Science, University of Southampton, Southampton, UK; 4 Escola de Artes Ciências e Humanidades, Universidade de São Paulo, São Paulo, Brazil

**Keywords:** Physical oceanography, Sedimentology, Geomorphology

## Abstract

Strong currents are a key component of benthic habitats by supplying food and nutrients to filter-feeding organisms such as cold-water corals. Although field measurements show that cold-water coral habitats exist in areas of elevated bottom currents, flume studies show that cold-water corals feed more effectively at lower flow speeds. This research aims to explore this disconnect in situ by utilising high spatial resolution ROV photogrammetric data coupled with high temporal resolution in situ acoustic doppler current profile measurements at seven study sites within the upper Porcupine Bank Canyon (uPBC), NE Atlantic. Object-based image analysis of photogrammetric data show that coral habitats vary considerably within the upper canyon. Although there is a regional hydrodynamic trend across the uPBC, this variation is likely driven locally by topographic steering. Although live coral tends not to face directly into the prevailing current direction, preferring lower local flows speeds, they can tolerate exposure to high-flow speeds of up to 114 cm s^−1^, the highest recorded in a *Desmophyllum pertusum* habitat. Not only do these high flow speeds supply food and nutrients, they may also help contribute to coral rubble production through physical erosion. These results can be incorporated into simulations of future deep-water habitat response to changing environmental conditions while extending the upper current speed threshold for cold-water corals.

## Introduction

Cold-water corals (CWC’s) such as *Desmophyllum pertusum* (recently synonymised from *Lophelia pertusa*^1^) and *Madrepora oculata* form three-dimensional, calcium carbonate skeletons that create frictional drag with the current, baffle sediments and create habitat for other organisms^2–12^. Continuous growth of such reef-building framework, accumulation of sediment and production of autochthonous coral rubble can lead to the development of a CWC reef or mound structure where all environmental conditions are suitable^6,7,13–19^. These structures range in height from < 5 m to > 100 m and range from tens of metres to several kilometres long^2,20–26^. CWC’s don’t always necessarily form reefs and mounds, they also generate habitats with accumulations of coral that do not generate topographic highs, so called CWC “gardens” or CWC “thickets”^27–29^. In either case, biodiversity and biomass in CWC habitats is significantly higher than in their surrounding areas^30^.

CWC habitats have been mapped to understand their occurrence, development and environmental tolerances in many parts of the world e.g. the Irish margin^22,31^, Gulf of Mexico^24,32^, the Scottish margin^33^, the Mediterranean^34^ and the Norwegian margin^9,35–37^. In many cases, sedimentary geomorphic features such as sediment waves and seabed scours exist in CWC habitats, demonstrating a correlation between CWC’s and intensified currents^21,38–40^. Clear examples evidencing this association can be found in Rebesco and Taviani^41^. This is supported by species distribution modelling studies that reveal in further detail that CWC distribution is influenced by current velocities^33,40,42–44^.

With progressively higher resolution mapping methodologies available (e.g. ROV- and AUV-mounted multibeam surveys), repeat mapping can be utilised to monitor the temporal dynamics in CWC habitats. Repeat side scan sonar mapping and ROV video data at the Darwin Mounds, NE Atlantic (UK waters) demonstrates the low-recovery rates of trawled CWC habitats over a period of 8 years^45^. Results from repeat imaging of the Piddington Mound the NE Atlantic (Irish waters) show that changes in biodiversity and sediment facies can be significant over 4 years^46,47^. As such, there is a need for local-scale CWC habitat research^48^.

Whilst repeat mapping occurs on longer time scales, gaining an understanding of the environment that CWC reside in requires in situ monitoring that achieves high resolution temporal data. Current speed data from various CWC habitats in the NE Atlantic, spanning periods 2 to 3 weeks, show that dense coral occurrences coincide with intensified bottom currents and further consider current regime an important CWC mound-building influence^49,50^. Longer term monitoring (up to 1 year) has shown that CWC’s live in energetic environments with high current speeds that may prevent sedimentation but provide sufficient food for corals to survive^8,51–57^.

CWC’s benefit from enhanced bottom currents. This is evidenced spatially by local scale seabed erosion or sediment transport features^58^ and temporally by in situ measurements^56^. Although current speeds of up to 60 cm s^−1^ have been measured at CWC’s habitats in the Gulf Stream^59^, Dorschel et al.^38^ and Huvenne et al.^21^ that at some threshold the current may inhibit CWC mound development At some upper current speed threshold however, the current may inhibit CWC mound development. In contrast to this, flume tank studies show that the corals themselves capture food more effectively at lower flow speeds^60,61^ and as such, quantified understanding of the effect of currents on CWC habitats is required. This research aims to quantitatively characterise several CWC habitats in the upper Porcupine Bank Canyon (uPBC), NE Atlantic and determine the effect of currents on their surface coverage (live, dead coral and coral rubble) using a unique combination of high resolution spatial and temporal datasets. Understanding the effect of currents on CWC status is essential to predict how they will respond to a changing climate^62^.

### Porcupine Bank Canyon

**Figure 1:**
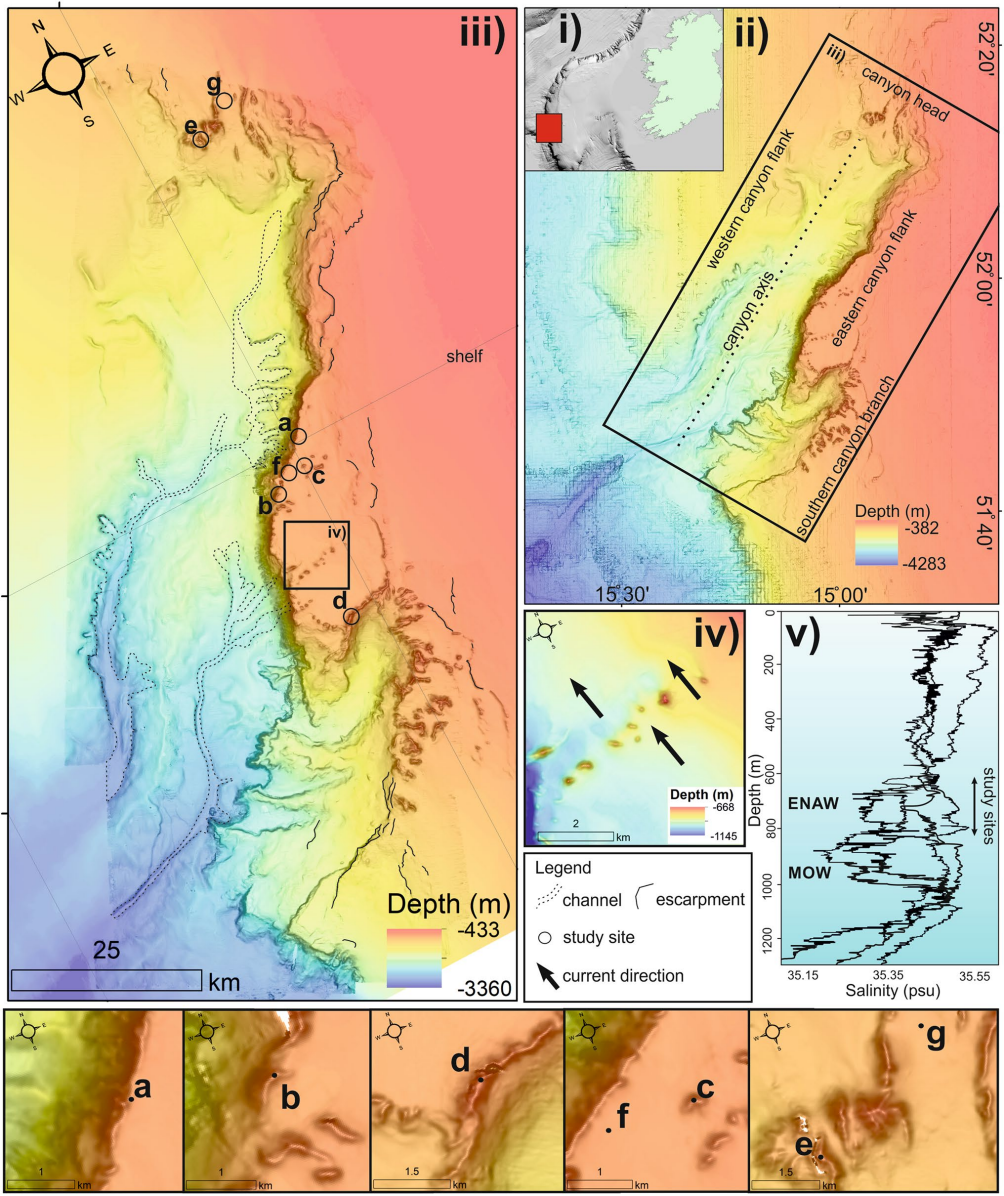
(**i**) map showing the location of the Porcupine Bank Canyon on the Irish continental shelf; (**ii**) bathymetric map showing the overall canyon geography; (**iii**) bathymetric map of the upper Porcupine Bank Canyon area showing main geomorphic features and study sites (**a**–**g**); (**iv**) close-up bathymetric data showing current-aligned scours in the lee of coral mound features; and (**v**) schematic adapted from Mazzini et al.^65^ showing salinity profiles and water masses within the upper Porcupine Bank Canyon. The depth distribution of study sites is indicated by a double-sided arrow within the ENAW. Maps created using ArcGIS Desktop v10.6 (www.arcgis.com).

The Porcupine Bank Canyon (Fig. 1) is a tectonically-controlled, north-east to south-west trending submarine canyon on the Irish-Atlantic margin^63,64^ with an inferred fault-scarp exposed along the eastern canyon flank in the upper canyon. Phosphatic-rich authigenic deposits indicate high biological productivity and low sedimentation rates^65^. The upper canyon exhibits CWC mound features between 550 and 900 m water depth, ranging from 50 to 200 m in height and 900 m to 2000 m in diameter^65,66^. Mound features are steep-sided and occur in clusters with a predominant north-west crest-alignment. The eastern canyon is dominated by compact sand and iceberg ploughmarks while mobile sand exists proximal to the mound clusters^65^. The main CWC-influencing water masses in the region at these depths are the warm, saline, northerly-flowing Eastern North Atlantic Water (ENAW) at 700 m water depth and Mediterranean Outflow Water (MOW) which can be characterised by a salinity high between 800 and 1000 m water depth and a permanent thermocline^40,67^. The NW Porcupine Bank is known for enhanced bottom currents^67^ which is also true locally on the shelf where sedimentary bedforms such as sediment drifts suggest that the prevailing current flows northward and is heavily directed by the local topography^65^.

## Materials and methods

Seven study sites were identified within the uPBC for this study (Fig. 1). Sites were chosen to encompass coral and non-coral habitats within the full geographic extent of the uPBC, as well as distinct seabed morphological features (e.g. mounds, ridges, flat areas and channels). This ensures that the research accurately represents the upper canyon.

### Multibeam bathymetry

Vessel-mounted multibeam echosounder data were collected over the uPBC area during the following research cruises on board the *RV Celtic Explorer*: QuERCi I (cruise number CE15009: Wheeler et al.^68^); CoCoHaCa II (cruise number CE18011: Lim et al.^69^) and; MoCha_Scan I (cruise number CE19008: Lim et al.^70^) imaging a total of 2055 km^2^. Data were acquired using a Kongsberg EM302 operated at a frequency of 30 kHz with a vessel survey speed of 8 knots. This achieved a swath width of approx. 2500 m. Positioning and attitude data were obtained using a C-Nav positioning system and Kongsberg SeaPath attitude sensor system respectively. Data acquisition was planned and managed within the SIS software suite, where calibration values, sensor offsets, real-time sound velocity, navigation and attitude data were incorporated. The MBES data were stored as *.all and *.wcd files and were processed and cleaned using QPS Qimera. The cleaned data were saved as a single *.xyz and gridded to a 25 m ArcView GRID. The gridded data were projected in UTM Zone 28 N and in ArcMap 10.4, slope angles (in degrees) were calculated from the bathymetric grid.

### Photogrammetry

ROV video data were acquired to create Structure-from-Motion (SfM) photogrammetric reconstructions of CWC habitats within the uPBC. All ROV video data were acquired during research cruises on the *RV Celtic Explorer* using the *Holland 1* ROV: MoCha_Scan I (cruise number CE19008:^70^) and; MoCha_Scan II (cruise number CE19014: Lim et al.^71^). At each study site (Fig. 1), high-definition video data (1080p) were acquired at 50 fps. During acquisition, using an altimeter, the ROV maintained a height of approx. 2 m above the seabed to achieve a consistent field of view. At > 2 m from the seabed, the ability to clearly identify coral was hampered due to lack of light while at heights of < 2 m from the seabed, ROV thrusters would occasionally cause sediment resuspension and provides a narrower field of view. These data were not included in the photogrammetric reconstruction. 12 lights ranging from 250 to 400 watts were attached to the ROV at a fixed angle in order to maintain consistent illumination within the field of view. ROV positioning data were recorded using a *Sonardyne Ranger 2* Ultra Short Baseline (USBL) beacon which were further corrected by an *IXBlue* Doppler Velocity Logger (DVL). In order to capture the 3D nature of the CWC frameworks, the camera was mounted at an oblique angle and the ROV manoeuvred in a spiral survey design to ensure high image overlap [e.g.^46^]. The ROV maintained a survey speed of approx. 0.4 knots. A set of laser scalers set at 10 cm apart, pointed at the field of view, were used as a scale reference. All video data were formatted and saved as *.mov.

Images were extracted at a rate of 1 image per second from the video data using Blender (v2.78). Image data for each site were imported to Agisoft Metashape. The process of generating a digital elevation model (DEM) and orthomosaic were carried out as specified by the Agisoft Metashape user manual and has shown accurate results in cold-water coral habitats^72^. Study-specific settings include “high-quality” for image alignment and “high-quality” for dense cloud reconstruction. Dense clouds were cleaned using the gradual selection toolbar and manual editing. Using a combination of laser-scales, USBL positioning and known sizes and positions within the images (e.g. the lander frame size and position), the dense clouds were scaled and georeferenced. Dense clouds were optimised and DEM’s and orthomosaics were generated at approx. 7 mm pixel resolution. DEM’s and orthomosaics were projected in UTM 28 N and saved as *.tif formatted images. Terrain variables (slope and aspect) were derived from the DEM’s using ArcGIS ToolBox.

### Marine object-based image analysis

Object-based Image Analysis (OBIA) is a widely used and automated image analysis methodology that involves 2 main steps: the grouping of image pixels into homogenous regions based on digital numbers into segments of meaningful information (segmentation) and; the assigning of these polygons/segments to specified classes (classification). The method has already been applied to CWC habitat photogrammetric data with successful results^73^. As such, segmentation and classification carried out here closely follow the methods of Conti et al.^73^. All data (DEM’s, orthomosaics and slope) were imported to eCognition Developer. A segmentation algorithm was applied to the imported dataset, with a scale parameter of 1400. Five classes were identified based on the aim of the study and classes identified by similar research^74^. The identified classes, henceforth termed facies, are Live Coral Framework (LCF), Dead Coral Framework (DCF), Soft Coral (*Leiopathes* sp.) (SC), Coral Rubble (CR) and Sediment (S). Each segment was individually inspected by an expert operator, who trained the data by manually assigning a minimum of 50 polygons to each class. The eCognition Nearest Neighbour classification tool was applied, which classified all segments based on their similarity to the training samples. The classified segments were manually inspected to ensure consistency of results. An accuracy assessment was carried out using eCognition’s accuracy assessment tool and a subset of training samples, which showed an overall accuracy of 75.5%. Aspect, which was derived from the DEM within ArcGIS ToolBox, shows the azimuthal direction of the steepest slope within the analysis window^75^. The segments which were classified as LCF were then used to extract the aspect values within these LCF areas. The resulting segments show the azimuthal direction that the coral colonies and branches are facing.

### Benthic monitoring

To measure temperature, current speed and direction at the study sites, a Nortek 1 MHz Aquadopp Acoustic Doppler Current Profiler (ADCP) was deployed at each site by ROV. ADCP’s were mounted on weighted negative buoyancy aluminium frames (“landers”). The landers were tested in a flume tank within a laboratory which showed that a current speed in excess of 110 cm s^−1^ was the threshold required to move the lander across a *frictionless* surface. The ADCP’s were upward-facing at approx. 1.5 m off the bottom and measured current speed (cm s s^−1^), direction (°), temperature (°C), tilt, pitch and roll at 10-min intervals ~ 1.4 m above the sensor head (~ 3 m above the bottom) from approx. May 15th 2019 to July 30th 2019. Current speeds can be measured from 0 to 10 m s^−1^ with an accuracy of ± 1% of measured value ± 0.5 cm s^−1^. Temperature can be measured from − 4 to + 40 °C with an accuracy of 0.1 °C. All ADCP data were imported to Nortek-specific ADCP processing software which removes low-quality data (only observed during deployment and retrieval periods) and anomalous data spikes. All data were added to an SQL database format where mean current speeds could be queried when the current was opposing the LCF segments aspect value at each study site (known as the coral-facing mean current speed). This differs from the mean current speed, which describes the overall mean current speed at that study site while the mean coral-facing current speed describes the mean current speed when the current is flowing directly towards the coral aspect.

## Results

### Canyon geomorphology

The bathymetric coverage (Fig. 1) reveals the PBC covers an area of 2055 km^2^ ranging from − 450 to − 3300 m water depth. The canyon has an asymmetrical morphology with a steeply sloping eastern canyon flank, believed to be tectonically controlled, and a gently sloping western canyon flank. There are a number of slope-incising channels near the base of the north, east and western canyon flanks. There are two main sinuous channels that exist within the canyon. Although both channels are broadly parallel to the canyon’s orientation, the westernmost of these channels is considerably wider and deeper. At the south of the eastern canyon flank, the southern canyon branch, a feeder canyon, exits into the main canyon. Where it intersects the main canyon, the slope has a ‘step-like’ morphology. The eastern canyon wall is considerably steep (approx. 60 to 70 degrees) and reaches up to 800 m in height and 35 km in length. Coral mound features exist between − 550 and − 900 m water depth at the canyon head (north), the eastern canyon flank and the southern canyon branch. In the north, coral mound features exist as a series of elongate canyon-parallel ridges that range from 50 to 250 m in height. On the eastern canyon flank, coral mounds form a 30 m to 50 m tall multi-summited ridges of coral carbonates along the canyon extending for approx. 30 km. In addition to this ridge of coral carbonate, elongate coral mounds exist along the shelf at the eastern canyon flank. Although these elongate mounds are not physically connected, they have a linear north-east to south-west distribution. Large scour pits exist on the northern, lee-side of coral mound features on the canyon shelf. The southern feeder canyon has a dense cluster of coral mounds along the canyon break. Mound features range from 60 to 200 m in height and are typically elongated in an east–west direction. In some cases, these elongated mounds appear to intersect forming a more complex morphology.

### Habitat characterisation

**Figure 2:**
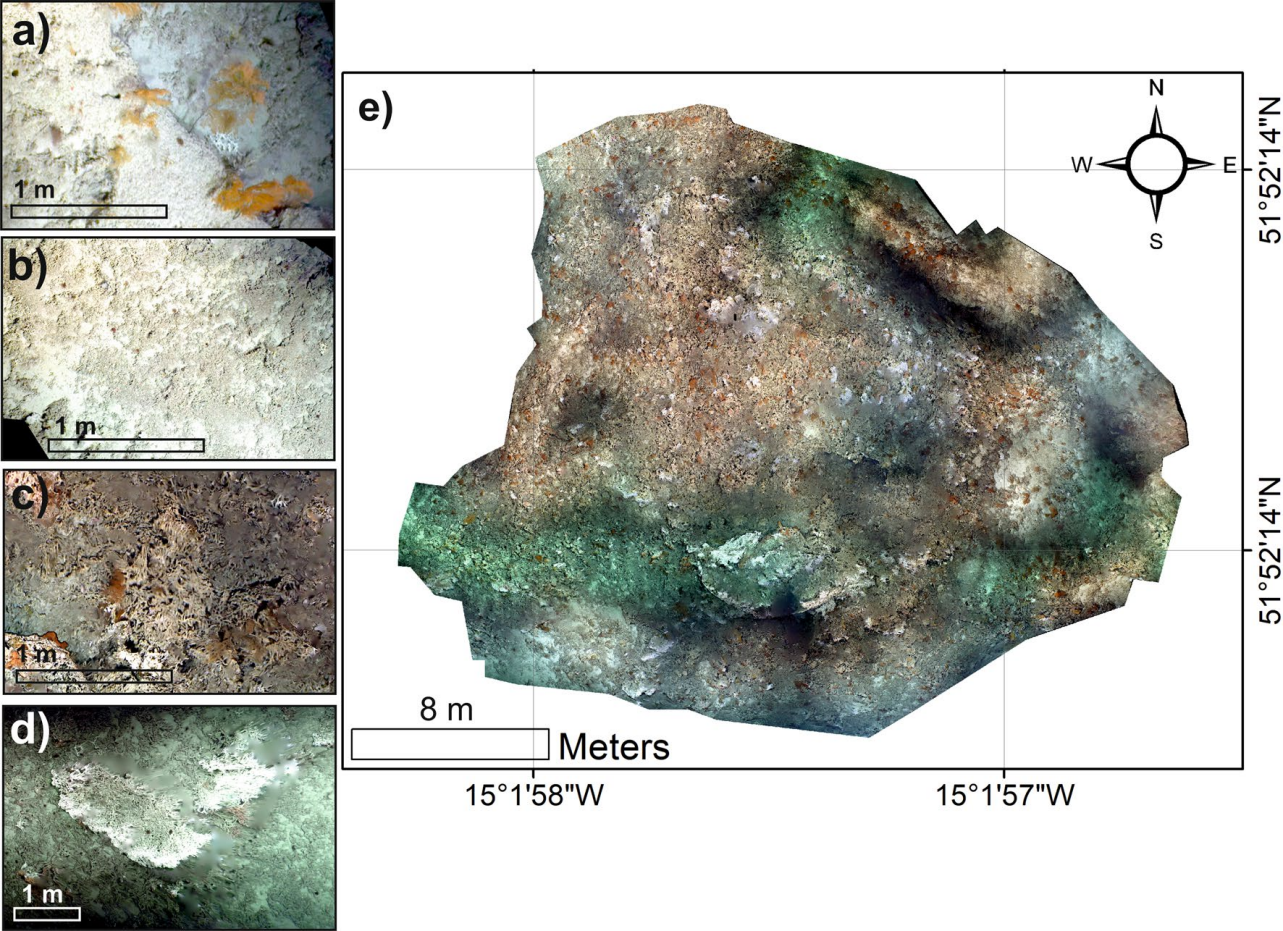
Example of an orthomosaic created in this study and example facies: (**a**) close up example image from the SC (*Leiopathes* sp.) facies; (**b**) close up example image from the CR facies; (**c**) close up example image from the DCF facies; (**d**) close up example image from the LCF facies; (**e**) example of the Study Site “d” orthomosaic. Maps created using ArcGIS Desktop v10.6 (www.arcgis.com).

A total of 7 reconstructions were generated utilising ROV-image data from each study site (Table 1, Fig. 2). Reconstructions are typically 76 m^2^ with the exception of the models from study site “c” and “d” which are 309 m^2^ and 703 m^2^ respectively. Range in reconstruction size in the coral areas is a result of the rejection of low-quality data around the edges of the models or insufficient image data to expand reconstruction range. An inspection of classification results showed no significant changes in results when reducing the reconstructions of models from study sites “c” and “d” to a similar sized area focussed around the immediate lander deployment spot within both study sites when compared to the full study site classification result.

**Table 1:** Model position, depth area and location within the canyon and live coral orientation.

Study site	Model reference	Latitude	Longitude	Depth (m)	Location	Coral presence	Live coral orientation
a	MS2_D3	52.004	− 14.98886	− 697	Eastern canyon flank	Yes	South east
b	MS1_D10	51.9735	− 15.04167	− 606	Eastern canyon flank	Yes	North east
c	MS1_D4	51.9832	− 14.999	− 645	Eastern canyon flank	Yes	North
d	MS1_D8	51.8701	− 15.03356	− 685	Southern canyon branch	Yes	North east
e	MS1_D2	52.2273	− 14.92565	− 719	Canyon head	Yes	West
f	MS1_D5	51.9837	− 15.01927	− 720	Eastern canyon flank	No	not applicable*
g	MS1_D1	52.244	− 14.87962	− 839	Canyon head	No	not applicable*

*not applicable as there is no coral at this location.

**Figure 3:**
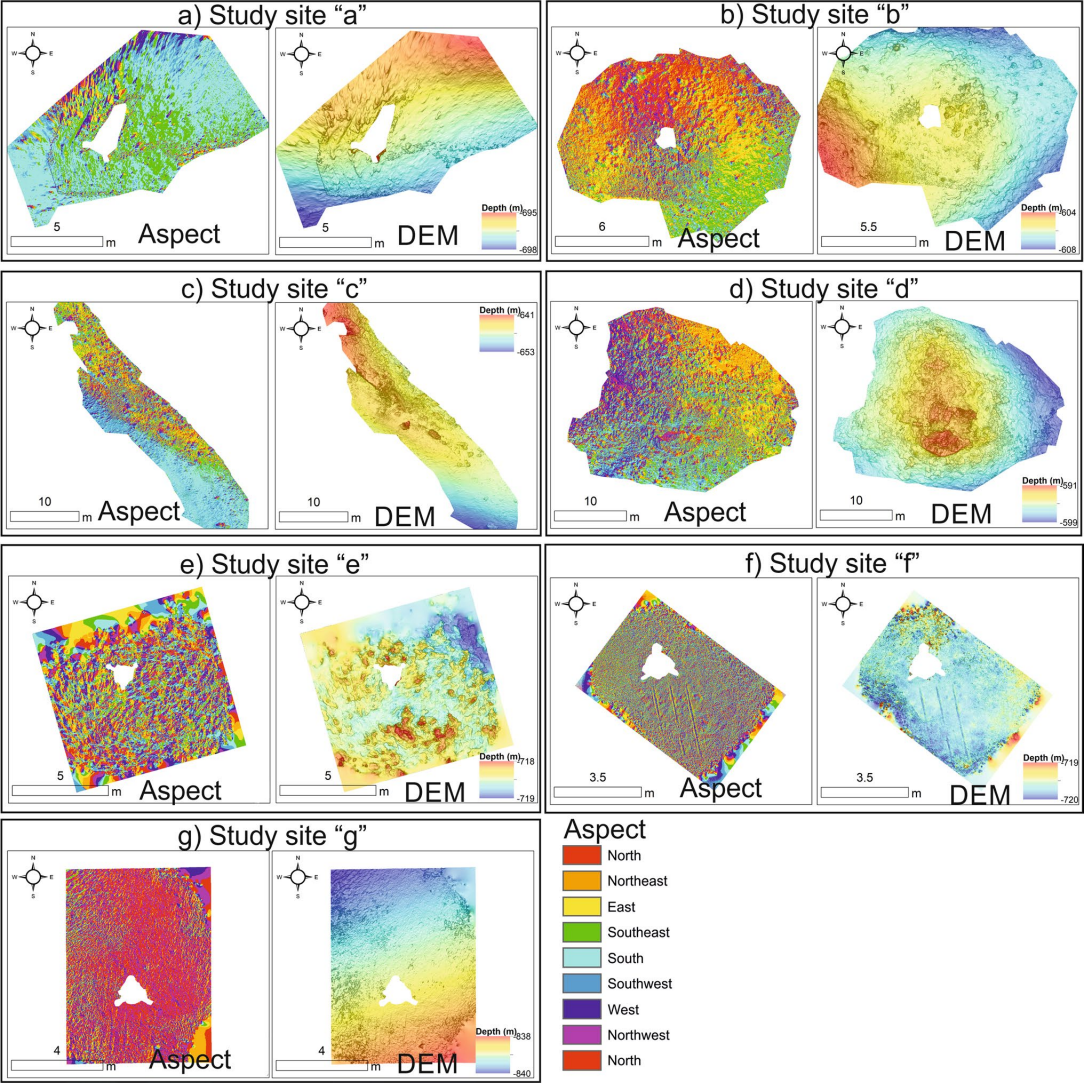
Digital elevation models (DEM’s) and aspect maps of each study site.

Derived digital elevation models (DEMs) and aspect maps for each study site are presented in Fig. 3. Descriptions of the DEMs and aspect maps are presented by study site below (see Sects. 3.2.1 to 3.2.7).

**Figure 4:**
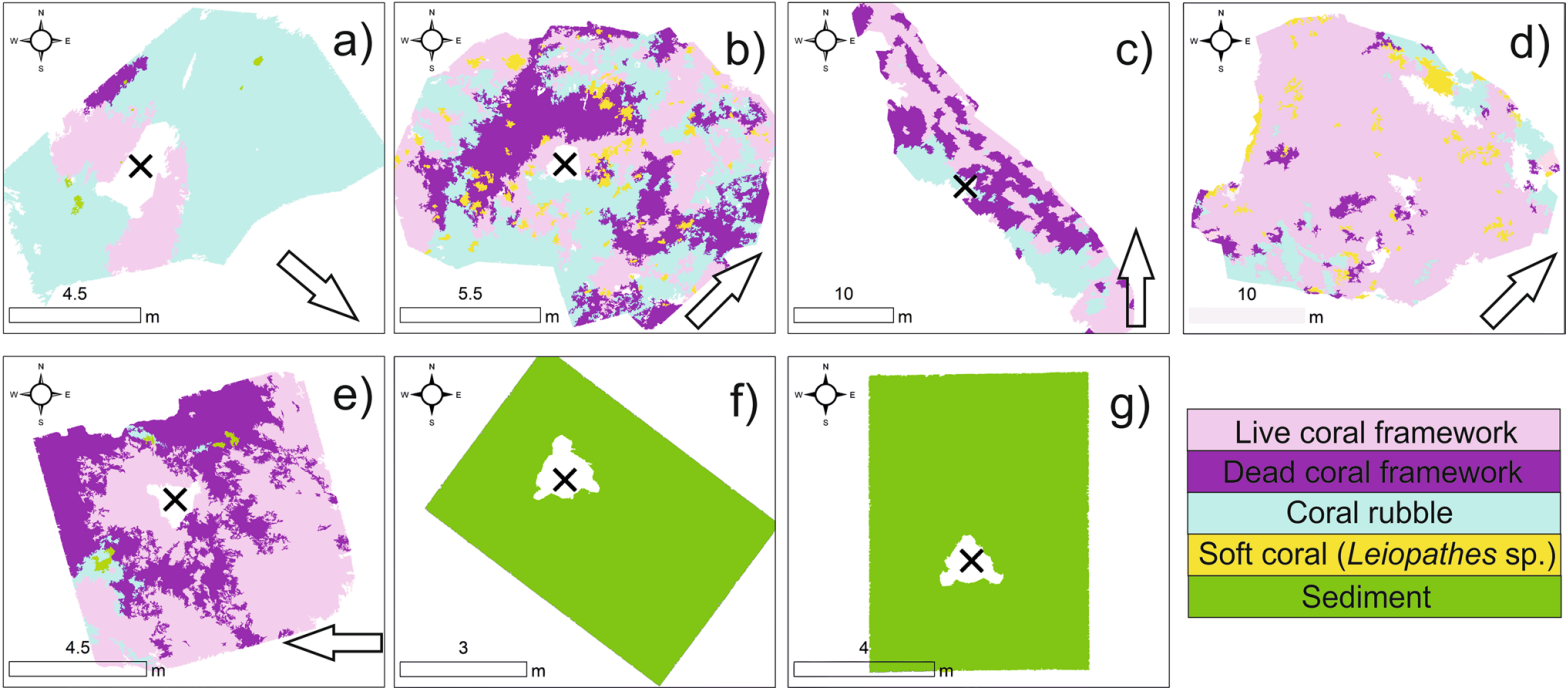
Object-based classification results of study sites (**a**–**g**) with the location of ADCP (x symbol) and aspect of the LCF facies (arrow). Maps created using ArcGIS Desktop v10.6 (www.arcgis.com).

**Figure 5:**
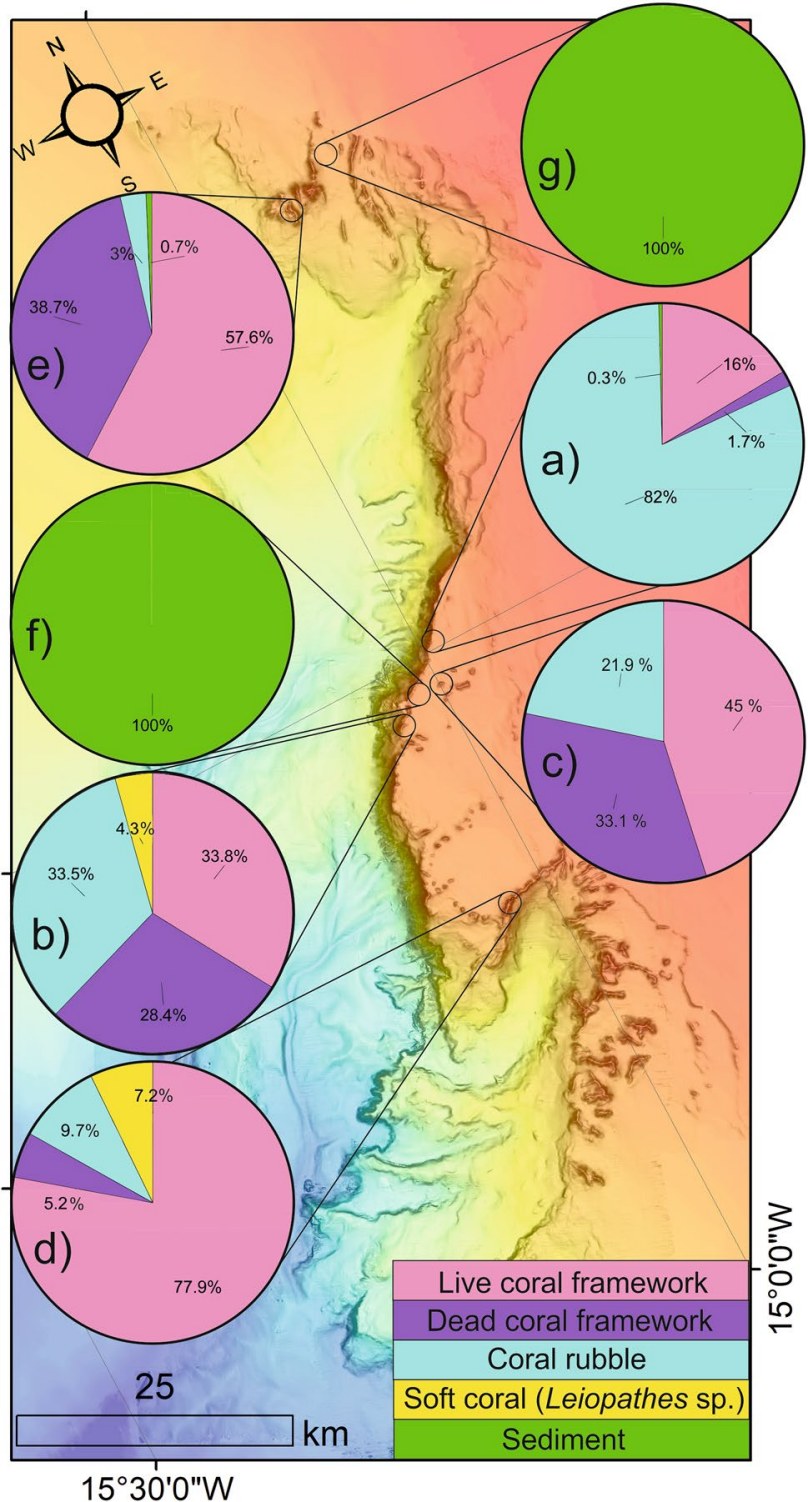
Map showing study site facies proportions created using ArcGIS Desktop v10.6 (www.arcgis.com).

Each study site orthomosaic is classified into facies using Object based Image Analysis (OBIA). The resultant map outputs are presented in Fig. 4 and their percentage facies composition is presented in Fig. 5. The data is descripted by study site below (see Sects. 3.2.1 to 3.2.7). Maps created using ArcGIS Desktop v10.6 (www.arcgis.com).

The seven ADCP’s monitored current speed, direction and temperature for 1 min every 10 min during the period spanning the 13th of May 2019 to 31st of July 2019. All lander-mounted ADCP’s were successfully retrieved within their original location with the exception of those deployed at study site “a” study site “b” where the landers had fallen over due to high current speeds during the deployment period. Using the attitude data stored within the ADCP raw data file, the time and date that the sensor fell over was determined and therefore only data from before this period were included in the study.

#### Study site “a”

**Figure 6:**
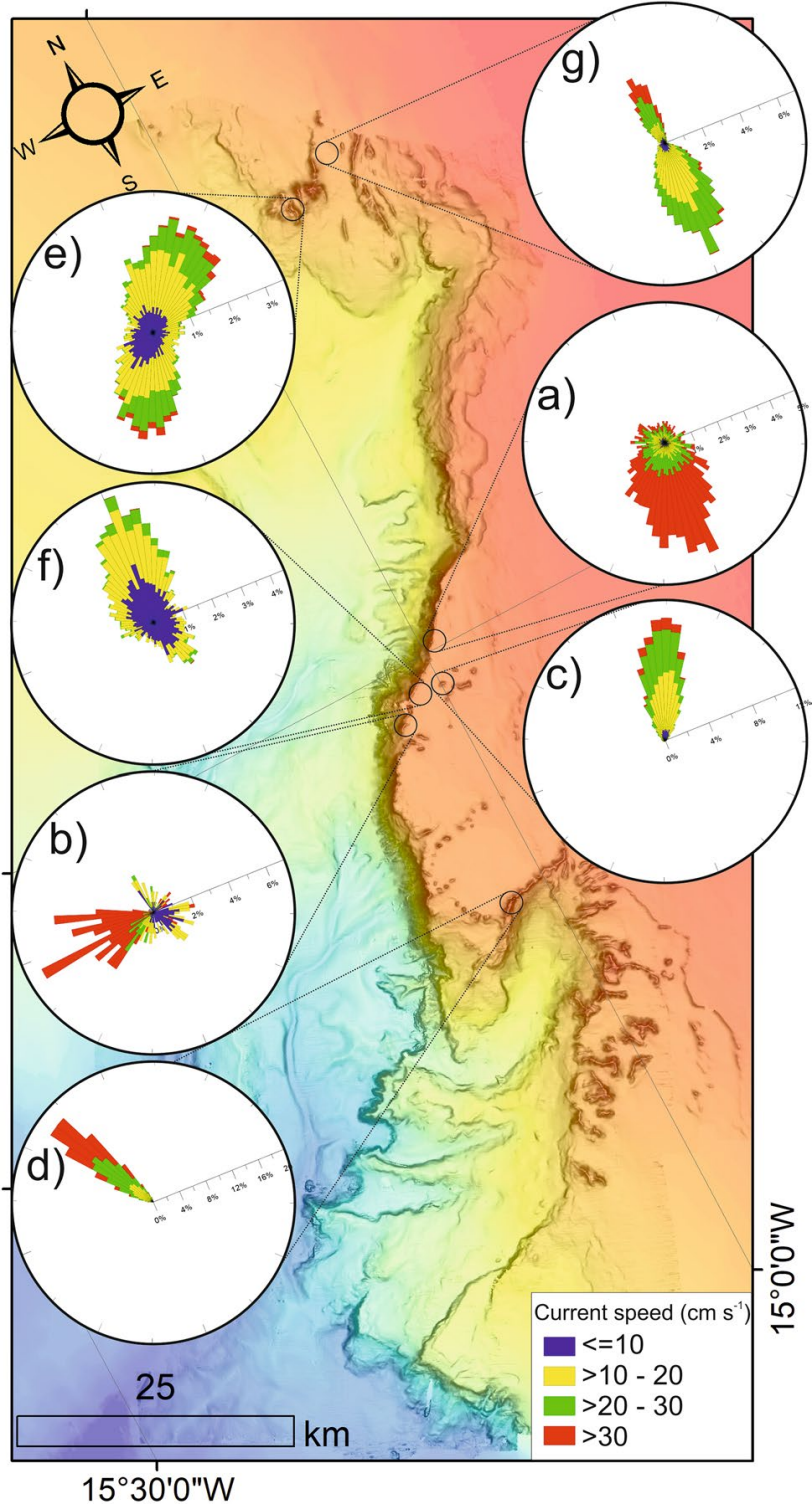
Map-orientated current speed rose diagrams. Map created using ArcGIS Desktop v10.6 (www.arcgis.com).

Study site “a” occurs near the edge of the eastern canyon flank (Fig. 1). It is part of a larger, approx. 30 m tall ridge feature that extends north–south, parallel to the canyon edge. At − 697 m water depth, the site covers an area of 85 m^2^ (Fig. 3). The most common facies is the CR facies (82%), the highest proportion of CR facies within the study (Figs. 4, 5). Soft corals (*Leiopathes* sp.) were not observed at the study site. The site ranges from − 695 to − 698 m (Fig. 3a) water depth and has an aspect predominantly south to south-east. Similarly, the LCF facies predominantly has an aspect to the south-east (Fig. 4; Table 1). The ADCP at study site “a” successfully recorded data for a period of 27 days, measuring a max current speed of 114.2 cm s^−1^, the strongest recorded from all the study sites, and average current speed of 31.3 cm s^−1^, also the highest, before falling over (Table 2). Currents here spend most time flowing in a certain direction where the flow > 30 cm s^−1^ (Fig. 6). The current flows at < 5 cm s^−1^ for 1.6% of the time, < 10 cm s^−1^ for 8% of the time and > 30 cm s^−1^ for 46% of the time. The effect of the tide is relatively low here, where the current flows predominantly in a southerly direction. The average recorded temperature during the deployment period is 9.4 °C (Table 2).

**Table 2:** ADCP data from each study site showing mean and max current speeds, the aspect of live coral frameworks, mean current speed when the current is flowing directly into these frameworks (‘coral-facing mean current speed’) and temperature data.

Study site	Dominant current direction (degrees)	Overall current speed (cm s^−1^)		Live coral orientation	Coral-facing mean current speed (cm s^−1^)	Temperature (°C)		
		Mean	Max			Mean	Min	Max
a	South (185)	31.3	114.2	South east	21.9	9.4	8.9	9.8
b	West (275)	24.0	75.9	North east	21.5	9.2	9.1	9.4
c	North east (40)	17.3	49.9	North	9.0	9.6	8.8	10.0
d	North west (325)	25.4	66.3	North east	7.0	9.6	8.6	10.1
e	North east (45)	9.4	55.7	West	8.0	9.4	8.5	9.9
f	North (5)	9.7	34.1	na	na	9.5	8.7	9.9
g	South (175)	18.2	56.4	na	na	8.6	7.7	12.3

#### Study site “b”

Study site “b” occurs near the edge of the eastern canyon flank (Fig. 1). It is part of the same ridge feature that study site “a” occurs on albeit considerably further south (10 km). However, study site “b” occurs near a break in the ridge feature. This break is orientated towards the canyon axis. At − 606 m water depth, the study site covers an area of 135 m^2^ (Fig. 3). It is characterised by approximately equal proportions of LCF facies (33.8%), DCF facies (28.3%) and CR facies (33.5%) (Figs. 4, 5). The site ranges from − 604 to − 608 m water depth (Fig. 3b). While the site has a range of aspect values, the LCF facies predominantly has an aspect to the north-east (Fig. 4; Table 1). The ADCP at study site “b” successfully recorded data for a period of 4 days, measuring a max current speed of 75.9 cm s^−1^ and average current speed of 24 cm s^−1^ (Table 2). Currents flow in all directions but the strong flows are predominantly in a westerly direction (Fig. 6). However, it flows in other directions with tidal flow. The current flows at < 5 cm s^−1^ for 5.4% of the time, < 10 cm s^−1^ for 28% of the time and > 30 cm s−^−1^ for 37% of the time. The average recorded temperature during the deployment period is 9.2 °C (Table 2).

#### Study site “c”

Study site “c” occurs on an isolated coral mound feature on the eastern canyon flank (Fig. 1). The mound is slightly elongate in an east to west orientation. It is 320 m in length and 80 m in height at its summit. Although it is surrounded by flat seabed, it is part of a cluster of other coral mounds. At − 645 m water depth, the study site covers an area of 309 m^2^ (Fig. 3). It is characterised by LCF facies (45%), DCF facies (33.1%) and CR facies (21.9%) (Figs. 4, 5). The site ranges from − 641 to − 643 m water depth (Fig. 3c) and has a typical aspect of south-east. The LCF facies typically have a northern aspect (Fig. 4; Table 1). The ADCP at study site “c” successfully recorded data for a period of 66 days, measuring a max current speed of 49.9 cm s^−1^ and average current speed of 17.3 cm s^−1^ (Table 2). Currents here predominantly flow in a north north-easterly direction with no apparent tidal influence (Fig. 6). The current flows at < 5 cm s^−1^ for 3.5% of the time, < 10 cm s^−1^ for 16.7% of the time and > 30 cm s^−1^ for 5% of the time. The average recorded temperature during the deployment period is 9.6 °C (Table 2).

#### Study site “d”

Study site “d” occurs on a north-east to south-west coral ridge feature along the southern feeder canyon ridge (Fig. 1). The ridge extends from the head of the southern canyon branch, along the edge of the northern flank for approx. 9.7 km. From the canyon head, it can be traced back to escarpment features along the southern canyon flank. At − 685 m water depth, the study site covers an area of 703 m^2^ (Fig. 3). It is predominantly covered by LCF facies (77.9%) but also has CR facies (9.63%), DCF facies (5.24%) and SC (*Leiopathes* sp.) facies (7.23%) (Figs. 3, 4). The site ranges from − 681 to − 689 m water depth (Fig. 3d). Although the site has a range of aspect values, the live coral has a predominant aspect to the north-east (Fig. 4; Table 1). The ADCP at study site “d” successfully recorded data for a period of 67 days, measuring a max current speed of 66.3 cm s^−1^ and average current speed of 25.4 cm s^−1^ (Table 2). Currents here predominantly flow in a north-westerly direction with no apparent tidal influence (Fig. 6). The current flows at < 5 cm s^−1^ for 1% of the time, < 10 cm s^−1^ for 6% of the time and > 30 cm s^−1^ for 31% of the time. The average recorded temperature during the deployment period is 9.6 °C (Table 2).

#### Study site “e”

Study site “e” occurs on a coral mound feature at the canyon head (Fig. 1). The mound has an “s-shaped” morphology, which is elongate from north to south. It is separated from a similar mound-like feature by a defined north–south channel. The mound is 1.3 km in length and 120 m in height. At − 719 m water depth, the study site covers an area of 73 m^2^ (Fig. 3). It is predominantly covered by LCF facies (57.6%) but also has CR facies (2.96%), DCF facies (38.7%) and S facies (0.65%) (Figs. 4, 5). The site ranges from − 718 to − 719 m water depth (Fig. 3e). Although the site has a range of aspect values, the LCF facies has a predominant western aspect (Fig. 4; Table 1). The ADCP at study site “e” successfully recorded data for a period of 66 days, measuring a max current speed of 55.7 cm s^−1^ and average current speed of 9.4 cm s^−1^, the lowest average recorded (Table 2). Currents here have a bi-modal orientation where the stronger flow (mode 1) is to the north-east and the slightly weaker flow (mode 2) is to the south-west, presumably related to tidal energy. The current flows at < 5 cm s^−1^ for 11% of the time, < 10 cm s^−1^ for 36% of the time and > 30 cm s^−1^ for 2% of the time. The average recorded temperature during the deployment period is 9.4 °C (Table 2).

#### Study site “f”

Study site “f” occurs in an off-mound setting on the eastern canyon flank ridge (Fig. 1). The seabed is largely flat and featureless, approximately 330 m north east from the canyon edge (Fig. 3). The area has previously been described as having compact or mobile sands (Mazzini et al.^65^). At − 720 m water depth, the study site covers an area of 34 m^2^ and is covered only by S facies (100%) with no coral (Figs. 4, 5). The site ranges from − 719 to − 720 m water depth (Fig. 3f). As the area is flat with no topographic expression, there is no prevailing aspect. The ADCP at study site “f” successfully recorded data for a period of 66 days, measuring a max current speed of 34.1 cm s^−1^, the weakest maximum current speed from all the study sites, and average current speed of 9.75 cm s^−1^ (Table 2). Currents here predominantly flow in a northerly direction but also flows to the south under the influence of the tide. The current flows at < 5 cm s^−1^ for 19% of the time, < 10 cm s^−1^ for 58% of the time and > 30 cm s^−1^ for < 0.1% of the time. The average recorded temperature during the deployment period is 9.4 °C (Table 2).

#### Study site “g”

Study site “g” occurs within a channel at the canyon head (Fig. 1). The channel is slightly bent from the north-east, exiting to the south, into the canyon head. It is flanked by ridge features on either side, which are parallel with the channel axis. At − 839 m water depth, the study site covers an area of 51 m^2^ (Fig. 3) and is only covered by S facies (100%) (Figs. 4, 5). The site ranges from − 838 to − 840 m water depth (Fig. 3g). As the area is flat with no topographic expression, there is no prevailing aspect. The ADCP at study site “g” successfully recorded data for a period of 66 days, measuring a max current speed of 56.4 cm s^−1^ and average current speed of 18.22 cm s^−1^ (Table 2). Currents here have a bi-modal orientation where the stronger flow (mode 1) is to the north and the weaker flow (mode 2) is to the south presumably related to tidal effects. The current flows at < 5 cm s^−1^ for 4% of the time, < 10 cm s^−1^ for 15% of the time and > 30 cm s^−1^ for 7% of the time. The average recorded temperature during the deployment period is 8.6 °C, the lowest mean temperature and also the site with the highest variation in temperature with a maximum temperature of 12.3 °C reached (Table 2).

### Discussion

This research presents the findings of 7 ADCP’s deployed in the uPBC over a period of approx. 75 days. At a depth range of − 606 to − 839 m and with a mean temperature range recorded during the deployment period of 8.6–9.6 °C, the study sites are located within the ENAW (Fig. 1; 700 m)^65^. At the Porcupine Bank Canyon, the ENAW regionally flows from the south to the north^65,76^. Large scour pits (Fig. 1) located on the northern, lee-side of coral mound features on the canyon shelf confirm this regional northerly current direction. The presence of such large scour pits likely reflect the long-term, net effect of the regional current in shaping the seabed. Moreover, the ADCP data from study site “f” (Fig. 6), where there is no topographic steering clearly demonstrates this flow direction. However not all study sites show this regional trend as considerable variation between current speed and direction at each study site suggests a strong local control. Currents deviate from this regional flow direction in the presence of topographic features such as the canyon ridge (study sites “a”, “b” and “d”), coral mounds (study sites “c” and “e”) and channels (study site “g”). The areas that deviate most from this regional trend are all located along the edge of the canyon (study sites “a”, “b” and “d”) (Fig. 6). These study sites also have the highest mean flow speeds (24–31.3 cm s^−1^). This localised increase in flow speed and deviation from the regional flow direction clearly demonstrates the ability of the canyon to steer and locally enhance flow speed^77^. The fact that study site “f” is only 330 m from the canyon with relatively low mean and max current speeds (9.7 cm s^−1^ and 34.1 cm s^−1^ respectively) and does not deviate from the regional trend suggests that although the influence of the canyon on hydrodynamics is not widespread, it is local and intense. It is also worth mentioning the scale of the observations within this study. Only study sites “f” and “g”, which were not proximal to local bathymetric highs, flow with the regionally northerly flowing ENAW. All other study sites, which are on or proximal to local bathymetric features, deviate from this direction. This is to be expected, given how close the ADCP’s are to the seabed where local obstacles will have a stronger impact.

Study site “c” which occurs on a coral mound at the canyon flank has a moderate mean current speed of 17.3 cm s^−1^ and flows directly to the north east (Fig. 6). This coral mound is likely generated by the growth of coral and subsequent baffling of current-suspended sediment, like many coral mound features on the Irish margin [e.g.^22^]. The deviation of the current from its regional northerly trend demonstrate the ability of the coral habitat to develop its own feedback cycle with the current.

The highest maximum current speeds recorded in the uPBC are 114 cm s^−1^ and 75.9 cm s^−1^ which occur at study sites “a” and “b” respectively, at the canyon ridge. Both ADCP lander frames fell over during the deployment period. During an initial sea acceptance trial of these systems, a current speed in excess of 110 cm s^−1^ was required to move these on a *frictionless* surface. Although the seabed was slightly sloping, the landers toppled upslope. As such, it is likely that both sites experienced current speeds greater than 110 cm s^−1^ to topple them.

Geophysical data shows that mound distribution in the uPBC is controlled largely by antecedent topography such as the canyon edges and escarpments^65^. Coral (study sites “a”, “b”, “c”, “d”, and “e”) and non-coral (study sites “f” and “g”) habitats are clearly different in terms of surface facies coverage (Fig. 5). Non-coral bearing habitats in this study are exclusively covered with sediment (100%), while the coral habitats have live and dead coral frameworks, coral rubble, soft coral and sediment. While coral habitats themselves are qualitatively similar, quantitatively, they exhibit considerable variation (Fig. 5). Habitats are either “coral rubble dominated” (> 50% CR) e.g. study site “a”, “live coral dominated” (> 50% LCF) e.g. study sites “d” and “e” or have mixed surfaces (all components < 50%) e.g. study sites “b” and “c”. All study sites recorded similar mean temperatures (8.6–9.6 °C) and exist within the same water mass (ENAW; Fig. 1). However, average current speeds in coral bearing habitats are notably higher (21.5 cm s^−1^) than in non-coral bearing habitats (13.9 cm s^−1^, and even 9.75 cm s^−1^ when excluding study site “g” which occurs within a channel at the canyon head which may be prone to higher current speeds). The variation in coral habitats (Fig. 5) does not follow a gradational spatial pattern across the canyon (i.e. changes from south to north). Local-scale hydrodynamics have previously been shown to produce clear variation in coral mound size and morphologies^20,39^ while local current direction has a significant influence on coral distribution^51^. As such, it is most likely that local hydrodynamic processes such as topographic steering influence habitat variability in the uPBC. This is consistent with previously reported research that shows that CWC habitats can be linked with enhanced bottom currents at various other locations in the NE Atlantic which can be attributed to food supply, sediment supply and waste removal^38,49,53,78,79^. However, whilst many studies have observed a relationship between coral and enhanced current speed, this research suggests that although coral habitats can survive at higher flow speeds, more coral grows in areas with lower flow speeds.

**Figure 7:**
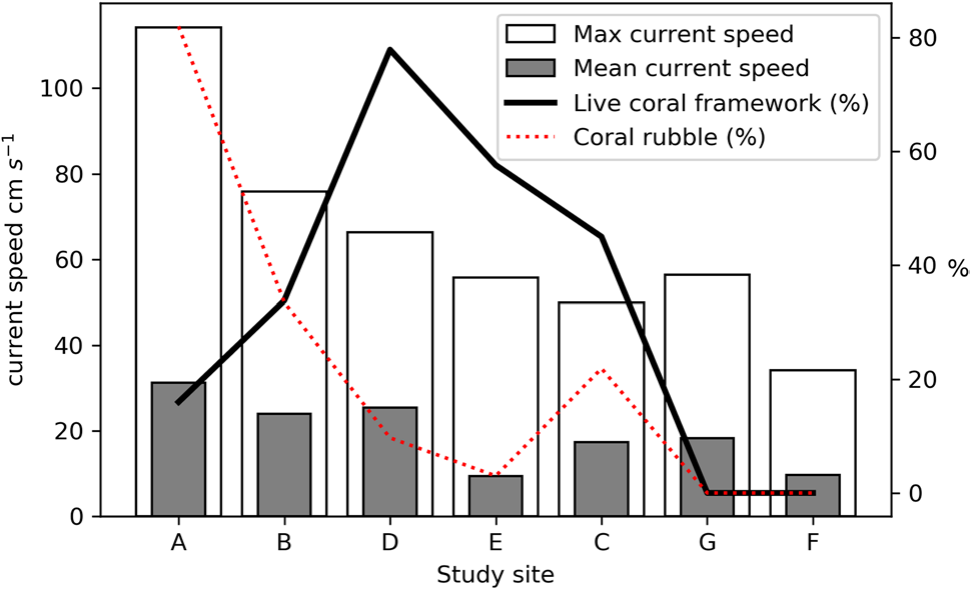
Facies proportion organised by maximum current speed.

There is a general trend where study sites with the highest proportions of coral rubble exist at progressively higher mean current speeds (Fig. 7). Coral rubble is typically produced through exposure at the surface for long periods which may lead to bioerosion or weakening of the coral framework^80^. With these measured high current speeds and evidence of mobile sands in the area^65^, it is likely that higher current speeds mean prolonged exposure of coral frameworks due to an inhibition of sedimentation. Thus, the increasing trend of coral rubble with increasing current speed may be explained by enhanced bioerosion weakening the frameworks and eventual physical erosion of the framework under high current speeds. The restricted cover of live coral at sites that were exposed to current speeds of 75 cm s^−1^ and beyond would also support this idea. Physical erosion of other CWC habitats by currents has been observed to expose underlying coral frameworks and produce coral rubble^46^. Dorschel et al.^38^ describe an “environmental window” for which CWC mounds develop: lower current speeds do not support dense accumulations of coral while excessively strong current speeds may hamper mound growth. A limitation of this interpretation is that the presence of more coral rubble at sites with higher current speeds may also be related to less sediment deposition which may then result in visually more coral rubble than at sites with lower current speeds.

**Figure 8:**
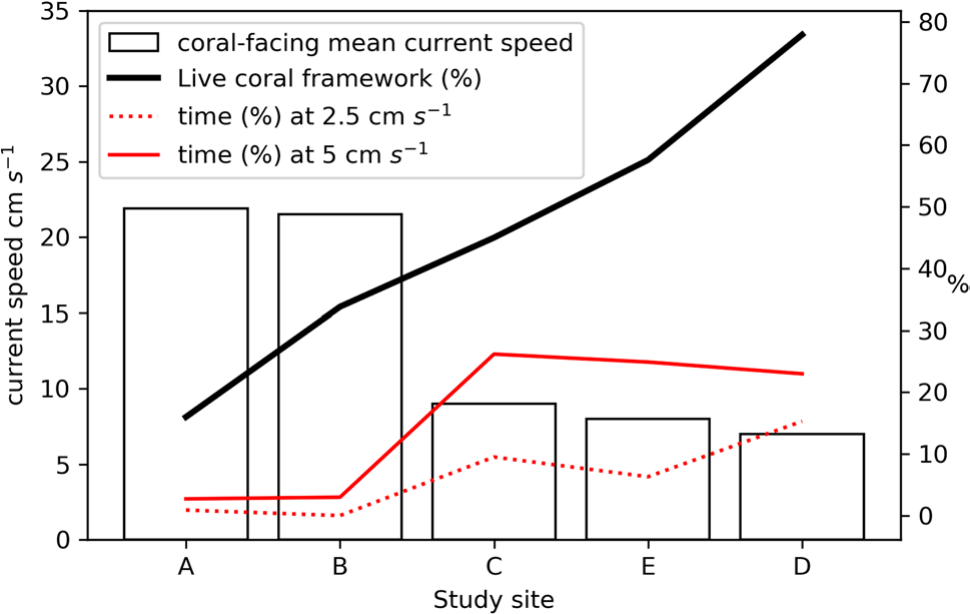
Live coral proportion organised by mean current speed when the current is flowing directly against the aspect of the coral based on currents facing the corals.

However, not all live coral frameworks face directly into the current (Table 2). When considering current flow velocity directly towards the live coral aspect, mean current speeds are lower, even up to 72% lower at study site “d” (from 25 to 7 cm s^−1^). Figure 8 shows the general trend of increasing live coral framework with decreasing current speed when the current is flowing directly into the aspect of the live coral. This may suggest that direct currents are too strong at some sites and thus sub-optimal for coral growth. Although previous studies [e.g.^8,21,38,49,55,59^] show that coral prefer enhanced bottom currents, both Purser et al.^61^ and Orejas et al.^60^ utilise flume tanks to show that corals capture food more effectively at lower flow speeds (zooplankton at 2.5 cm s^−1^ and phytoplankton at 5 cm s^−1^ in Orejas et al.^60^ only) with polyp expansion greatest at speeds of 0.5–6.7 cm s^−1^. Mortensen et al.^37^ noted that coral polyps exposed to a higher flow velocity in aquaria had a higher mortality rate. In support of these aquaria studies, this research provides in situ field evidence showing that the live coral did not face directly into the prevailing current. Moreover, when the current does flow directly towards the live coral colonies and branches, larger quantities of live coral exist at the sites with lower coral-facing mean current speeds. This is consistent with other parts of the Irish margin where although the CWC habitats exist in areas with high peak current speeds, on average the currents range between 10 and 15 cm s^−1^^8^. Furthermore, live coral has been observed growing on the current-sheltered lee-slope of coral mounds (e.g. the Piddington mound and the Hedge mounds)^38,74^. However, this is not always the case as coral has also been reported on mound summits e.g. Dorschel et al.^49^ as well as the current-facing side of the mound structure^36,53,81^ at other locations. Figure 8 also shows the percentage of time the current flows toward the coral aspect at speeds (2.5 cm s^−1^ and 5 cm s^−1^) identified by Orejas et al.^60^ at which food capture is most effective. This shows a trend of increasing LCF with increasing percentage of time at which the current flows towards the coral at those food capture effective speeds. For the reasons above, it appears that the corals favour lower flow speeds but can survive at higher flow speeds. However, it is important to note that Purser et al.^61^ and Orejas et al.^60^ are laboratory experiments that have been completed in flume tanks with single colonies and may behave differently from a reef structure. It is also known from cold as well as tropical corals that the 3D structure will influence flow on local scales which can influence flow patterns as well as influence feeding behaviour and sedimentation patterns. Furthermore, living prey were used in both flume experiments whose swimming capacity may also increase the difficulty to prey capture during high flow speeds^60^. In addition, it is still unclear what the corals are likely feeding on in the PBC. Moreover, as the laboratory studies have been completed in flume tanks, the current is unidirectional and will not change during tidal cycles. As such, comparison between the flume study and in situ results presented herein should be made with caution.

Many of the study sites show a tidal signature (a, b, e, f, g; Fig. 6). This is likely a semi-diurnal tidal current which has been measured at this side of the Rockall Trough^8^. These tidal currents may be of extreme importance to the corals and their orientation (growth direction). Firstly, the reduced velocity tidal ebb flow may contribute to lower flow speeds where there are otherwise higher flow speeds. This is especially true given that the site with the highest proportion of LCF (“d”) only flows at < 5 cm s^−1^ at < 1% of the time. Secondly, given the effect of local topography near the bottom where the corals grow, the change of flow direction may expose other parts of the reef to the current which may be otherwise sheltered. However, current speed and direction data from several parts of one CWC habitat would need to be acquired to confirm this.

While the corals here may feed at lower mean current speeds, overall speeds reported here show that live coral can survive exposure to notably high speeds (114 cm s^−1^ at study site “a”; 75.9 cm s^−1^ at study site “b” and 66.3 cm s^−1^ at study site “d”) although presumably feeding during short window of slack water as the tide turns. In fact, at 114 cm s^−1^, this is the highest recorded current speed recorded for live CWC habitat. At Tisler reef, offshore Norway, current speeds of 99.8 cm s^−1^ have also been recorded^51^. The prevalence of CWC at sites where current velocities exceed their natural range to efficiently feed, is likely supported by the baffling of flow velocity by the 3D framework^82^.

*Leiopathes* sp. does not occur at all study sites (study sites “b” and “d” only). These sites have a mean current speed of 24 and 25.4 cm s^−1^ (maximum of 75 and 66 cm s^−1^) respectively and are the only study sites to occur within this range.

The results of this research provide a number of unique, wider-scale implications. The time, cost and resource-intensive nature of sampling and surveying the deep-ocean make it uneconomically viable to map out the distribution of all cold-water coral habitats. Thus, predictive habitat suitability modelling is required and have previously been applied to cold-water coral habitats using terrain and environmental data in the north east Atlantic^83^, south Pacific oceans^84^ and on a global scale^85^. Pearman et al.^42^ and Bargain et al.^43^ show that inclusion of hydrographic variables such as current speed in predictive mapping clearly improves model performance. Our dataset endorses the inclusion of high-resolution hydrodynamic variables to improve predictive model performance, as clear linkages between flow strength and reef surface coverage (live, dead coral and coral rubble) was observed. The current speeds recorded in this study and, in particular, the highest current speed recorded within a CWC habitat (114 cm s^−1^), can be used to produce more accurate cold-water coral habitat suitability maps from local to regional scales through new knowledge of their distribution range.

An increasing trend in the global oceanic kinetic energy since the 1990′s associated with greenhouse warming indicates that ~ 76% of the upper 2000 m in the global ocean will incur a ‘substantial’ acceleration in global mean ocean circulation^62^. The results herein not only show that CWC habitats can tolerate considerably high current speeds, but that the results may provide a baseline/control for which CWC habitats may respond should current speeds increase under changing climate scenarios. We speculate that changes in the flow velocity at local sites, induced by larger climatic driven changes in circulation will have implications on CWC habitat composition, for example leading to environmental changes in favour of rubble production or live coral growth. This likely has implications for reef development and subsequently reef associated communities.

Whilst many studies show a relationship between coral and enhanced current speed, this research shows this relationship for the first time at a fine spatial scale (cm) using a quantified classification of high-resolution, photogrammetric data (SfM DEM’s and Orthomosaics) from a range of study sites within one segment of a submarine canyon. Such accurate quantification of coral cover can only be achieved by imaging large areas. This showcases the benefit of undertaking SfM surveys to quantify immediate habitat coverage and coral orientation, supplementing temporal current speed and direction datasets to build a holistic understanding of the local environment. Whilst data from five coral sites do not provide conclusive evidence, the trends are considerably strong and provide further insight into the finer-scale relationship between coral cover and flow velocity.

## Conclusions

Results presented herein show that, although the ENAW flows northerly over the uPBC, locally it is steered and intensified by topographic expression. Combining Structure-from-motion outputs and Marine object-based image analysis allows for accurate classification of cold-water coral habitats. While qualitative analysis of these results shows differences in coral and non-coral habitats, only quantitative analysis shows variation between coral habitats. Analysis of 3D photogrammetric reconstructions coupled with benthic current data shows that live coral tends to not face directly into the current. When the current does flow directly towards the corals, sites with lower flows speeds tend to have larger quantities of live coral, in line with flume tank observations. In situ current speeds show that live coral can exist in current speeds up to 114 cm s^−1^, higher than any other reported current speed in a CWC habitat. The high current speeds are also important in generating coral rubble and exposing adding to habitat heterogeneity. This research represents an example of the benefits in utilising high-spatial and temporal resolution datasets in deep marine research. Results herein can be used to increase the performance of predictive habitat suitability models. They can also serve as a control for how cold-water coral habitats may respond to changing deep water environmental conditions.
